# Myotendinous junction adaptations to ladder-based resistance training: identification of a new telocyte niche

**DOI:** 10.1038/s41598-020-70971-6

**Published:** 2020-08-24

**Authors:** Jurandyr Pimentel Neto, Lara Caetano Rocha, Gabriela Klein Barbosa, Carolina dos Santos Jacob, Walter Krause Neto, Ii-sei Watanabe, Adriano Polican Ciena

**Affiliations:** 1 Laboratory of Morphology and Physical Activity (LAMAF), Institute of Biosciences (IB), São Paulo State University “Júlio de Mesquita Filho”-UNESP, Rio Claro, SP Brazil; 2 Laboratory of Morphoquantitative Studies and Immunohistochemistry, Department of Physical Education, São Judas Tadeu University (USJT), São Paulo, SP Brazil; 3 Department of Anatomy,ICB-III, University of São Paulo-USP, São Paulo, SP Brazil

**Keywords:** Cell adhesion, Cell migration, Cell signalling, Anatomy

## Abstract

The present study shows chronic adjustments in the myotendinous junction (MTJ) in response to different ladder-based resistance training (LRT) protocols. Thirty adult male *Wistar* rats were divided into groups: sedentary (S), calisthenics (LRT without additional load [C]), and resistance-trained (LRT with extra weight [R]). We demonstrated longer lengths of sarcoplasmatic invaginations in the trained groups; however, evaginations were seen mainly in group R. We showed a greater thickness of sarcoplasmatic invaginations in groups C and R, in addition to greater evaginations in R. We also observed thinner basal lamina in trained groups. The support collagen layer (SCL) adjacent to the MTJ and the diameters of the transverse fibrils were larger in R. We also discovered a niche of telocytes in the MTJ with electron micrographs of the plantar muscle and with immunostaining with CD34+ in the gastrocnemius muscle near the blood vessels and pericytes. We concluded that the continuous adjustments in the MTJ ultrastructure were the result of tissue plasticity induced by LRT, which is causally related to muscle hypertrophy and, consequently, to the remodeling of the contact interface. Also, we reveal the existence of a collagen layer adjacent to MTJ and discover a new micro anatomic location of telocytes.

## Introduction

The myotendinous junction (MTJ) consists of a highly specific anatomical region in which the sarcoplasmatic membranes connect to bundles of extracellular matrix (ECM) collagen fibers^1^. Due to its functionality, MTJ represents the region with the highest transmission of force^2,3^.

Morphologically, MTJ presents projections that penetrate the muscle tissue, parallel and directed to the myofibrils, forming sarcoplasmatic invaginations. Currently, we know that its development is mainly influenced by the interactions between myoblasts and ECM elements^4,5^. The basal lamina of muscle fiber creates a supramolecular connection structure composed of different levels of proteins from the laminin and collagen polymers located in the ECM^6^. This arrangement contributes structurally to the transmission of force, adapting, remodeling, and consequently affecting the macroscopic tendon structure to different stimuli^7,8^.

Initially, telocytes were described as interstitial Cajal cells^9^. Telocytes are interstitial cells of stromal origin with an oval shape, and a heterochromatic nucleus with moniliform projections denominated telopodes, which characterize its morphological classification according to the number of projections^9,10^. They are founded in the interstitium of tissues such as the testicle^9^, smooth and cardiac muscle^11^, human tongue^10^, pancreas^12^, and liver^13^. It can be identified by the electronic microscopy, immunohistochemistry, and CD34+/immunostaining^14^. Recently, telocytes have been found in proximity to and engaging in possible interactions with satellite cells^10^. The telocytes projections have terminals (pods) with mitochondria that form junctions with adjacent cells, and they have caveolae that perform exocytosis through communicating vesicles (exosomes) to affect associated tissues^14^.

The MTJ interface is dynamic and highly complex, being susceptible to ultrastructural adaptations to different stimuli^15,16^. Trauma at the muscle–tendon interface leads to functional reductions in tissue properties, in addition to contributing to future injuries associated with high demand for sports training or physical inactivity^8,17^. The risk of trauma due to muscle tension is probably a confluence of numerous factors related to the MTJ's ultrastructural architecture and its contact surface^18,19^. Thus, physical training appears as an alternative to strengthen the muscle–tendon structure and ensure an effective transmission of force from the skeletal muscle to the tendon^20–23^.

According to Curzi et al., MTJ plasticity seems to be related to exercise intensity^24^. Jakobsen et al. showed that 4 weeks of resistance training (RT) could stimulate protection against stress injuries in the tendon^20^. Besides, Geremia et al. demonstrated that tendon hypertrophy induced by high RT intensity contributed to an additional increase in tendon stiffness^21^. Also, the increase in tendon stiffness appears to be caused mainly by adaptations in the properties of tissues. However, understanding of the composition of MTJ and the ability to adapt to loading is still weak.

Here, we reveal the presence of a support collagen layer (SCL) adjacent to MTJ Unexpectedly, we describe the existence and microanatomical location of telocytes in this region for the first time, considered the central area of force transmission, and susceptible to more severe mechanical damage.

## Results

### Plantaris muscle mass

**Figure 1:**
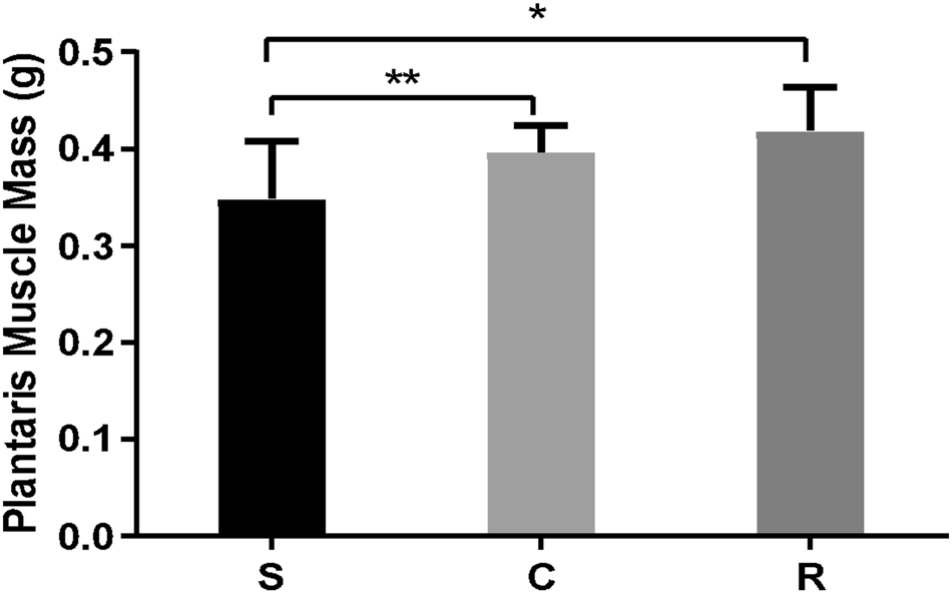
Means and standard deviations of the plantaris muscle mass of the Sedentary (S), Calisthenics (C), and Resistance-trained (R). Legend: S ≠ C **(p < 0.05); S ≠ R *(p < 0.005).

In groups, C and R, the mass of the plantaris muscle was 13% (p < 0.05) and 20% (p < 0.005) greater than S, respectively (Fig. 1).

### Myotendinous junction plasticity

**Figure 2:**
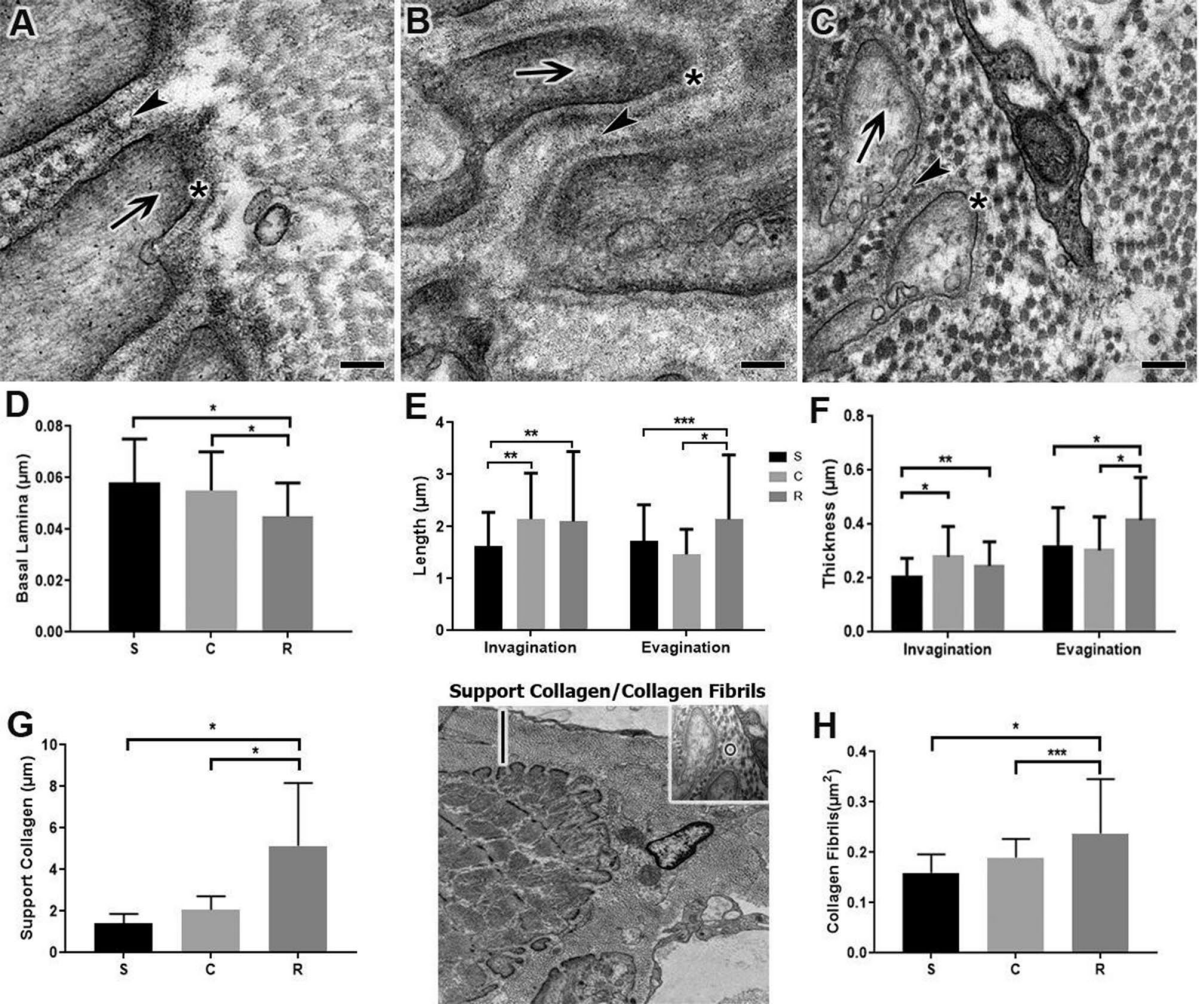
Transmission electron micrographs of the surface of the myotendinous junction reveal the sarcoplasmatic evaginations (arrows) interacting with the sarcoplasmatic invaginations (arrowheads) of the Sedentary (**A**), Calisthenic (**B**) and Resistance (**C**) groups. Scale Bar: 1 μm. Magnifications: 80,000× (**A**–**C**). (**D**) Means ± SD of basal lamina (*) thickness *(p < 0.0001). (**E**) Means ± SD of the lengths of the sarcoplasmatic invaginations and evaginations *(p < 0.0001), **(p < 0.001), ***(p < 0.01). (**F**) Means ± SD of invagination and sarcoplasmatic evagination thickness *(p < 0.0001), **(p < 0.001). Transmission micrograph identifying the light bar on the MTJ and highlighting the diameter representation of collagen fiber in this region (circle). (**G**) Means ± SD of the support collagen thickness *(p < 0.0001). (**H**) Means ± SD of the collagen fibril diameter in the MTJ *(p < 0.0001), ***(p < 0.01). Support collagen/collagen fibrils. The micrographs show the definition of the thickness of the support of the collagen fibrils layer adjacent to the MTJ and prominently positioned lengthwise in the layer of support where it was possible to measure its diameter.

In the experimental groups, the thickness of the basal lamina adjacent to the MTJ was measured, and the changes were related to the training of the groups (Fig. 2A–C). Group R showed a thickness of 22% thinner than S (p < 0.0001). Among the trained groups, the basal lamina thickness of group C was 18% thicker than R (p < 0.0001) (Fig. 2D).

In S, the region of interaction between the muscle cells and the connective tissue of the ECM revealed thin sarcoplasmatic invaginations in the intersection with the conical-shaped sarcoplasmatic evaginations, consisting of protein myofilament bundles, the distal sarcomeres (Fig. 2A).

In C, we observed long, and thick sarcoplasmatic invaginations composed of collagen fibers that were longitudinally arranged near the contact surface. At the end of the muscle cell, sarcoplasmatic evaginations that interacted with the tendon tissue were formed (Fig. 2B). The lengths of the sarcoplasmatic invaginations in C were 32% (p < 0.0001) longer than S (Fig. 2E). The invagination thickness of C relative to S was increased by 36% (p < 0.0001, Fig. 2F).

In R, it was possible to highlight the longitudinal bundles of collagen fibrils that were coming from the ECM of the sarcoplasmatic invaginations and interacting with the evaginations, demonstrating the remodeling of the ECM and the great adaptations in the MTJ (Fig. 2C). In R, sarcoplasmatic invaginations and evaginations were 29% (p < 0.0001) and 23% (p < 0.05) greater in length compared to S, respectively. In C group, invagination length was 2% greater than R (p < 0.05). Also, in R group, we showed that sarcoplasmatic evaginations length was 45% greater than C (p < 0.0001, Fig. 2E).

Regarding thickness, in R there was an increase of 20% (p < 0.005) in invaginations compared to S, and a reduction of 12% (p < 0.01) compared to C, while for sarcoplasmatic evaginations compared with S there was an increase of 31% (p < 0.0001), and compared to C, there was an increase of 37% (p < 0.0001) (Fig. 2F).

In all experimental groups, the SCL adjacent to the MTJ was identified, with its transversely arranged collagen fibrils and their alterations. The thickness of the collagen backing layer in R was 256% and 149% thicker compared to S and C, respectively (p < 0.0001, Fig. 2G). Regarding the diameter of the transverse collagen fibrils, C showed an increase of 18% relative to S (p > 0.05). In comparison, in R, the increase was 48% (p < 0.0001) relative to S. Between the trained groups, R showed a 25% greater diameter (p < 0.01, Fig. 2H).

### Telocytes

At the MTJ of three experimental groups, telocytes were identified with intimate relationships at the interface through their telopodes. They had close relations with the sarcoplasmatic invaginations, the basal lamina, the blood capillaries, the satellite cells, and the pericytes.

**Figure 3:**
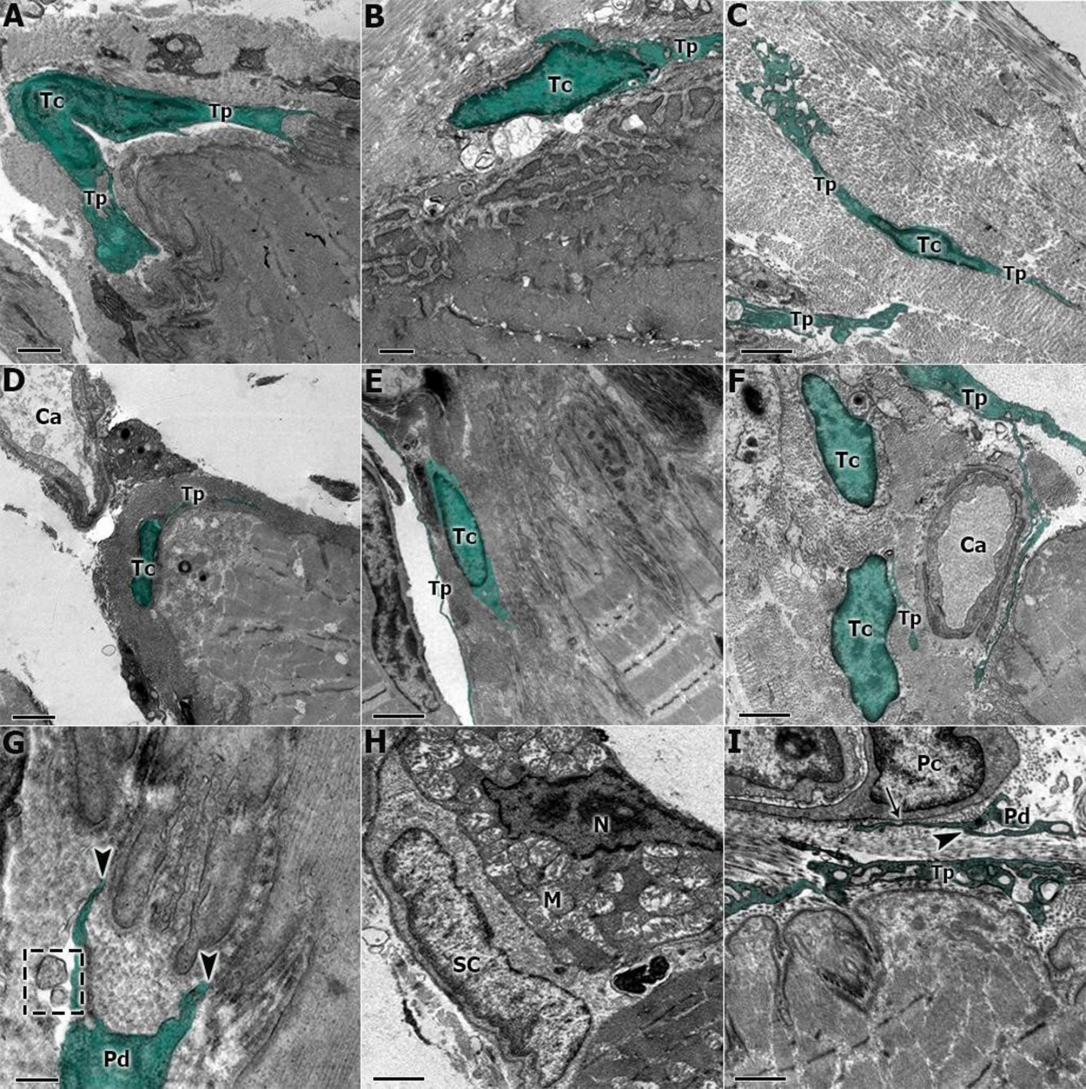
(**A**) The transmission electron micrograph of S shows the telocyte (Tc) adjacent to the MTJ and the telopodes (Tp). (**B**) The C group shows the ultrastructural adaptations of the MTJ and the interactions of the telocytes (Tc) in this region. (**C**) In the R group, we can observe a telocyte (Tc) with your telopodes (Tp) in proximity with the MTJ interface and surrounded by transversal collagen fibrils. (**D**) In S, we found evidence of capillaries (Ca) associated with telocytes (Tc) adjacent to the myotendinous region. (**E**) In C, the interactions of the telopodes (Tp) and telocytes were observed in the support collagen region associated with the MTJ. (**F**) Telocytes (Tc) adjacent to the blood capillary (Ca) in the MTJ. (**G**) In the S group at higher magnification; it is possible to observe the activity and contact between the telopodes terminals (Pd) that possible perform a paracrine activity with the sarcoplasmatic invaginations of the MTJ (arrowhead) and the vesicles released in this region (square). (**H**) In the C group it was possible to visualize the nucleus of a muscle cell (N), mitochondrial clusters (M), and a satellite cell (SC) in the MTJ. (**I**) At higher magnification, the communication junction (arrow) between telopodes (Tp) and the pods with pericytes (Pc) (arrowhead) in group R. Scale Bar: 1 µm (**A**,**B**,**D**,**F**); 2 µm (**C**,**E**,**H**,**I**) 0.2 µm (**G**). Magnifications: 4,500× (**E**); 5,000× (**C**,**D**); 6,000× (**F**); 8,000× (**B**); 10,000× (**A**); 15,000× (**H**,**I**), 40,000× (**G**).

In S, telocytes were found in the ECM adjacent to the MTJ region, and their telopodes were interacting with the sarcoplasmatic invaginations and the SCL surrounding the MTJ (Fig. 3A). Blood capillaries and telocytes adjacent to the MTJ were observed (Fig. 3D). In higher magnification, we can identify the possible paracrine activity of the pods and the release of vesicles in the MTJ region (Fig. 3G).

In C, telocytes were also observed adjacent to the MTJ and their extensions were associated with the sarcoplasmatic invaginations (Fig. 3B). Also, in C, we found a telocyte and its telopodes related to the collagen layer between the tendon and the MTJ (Fig. 3E). In addition, we noted the subsarcolemmal mitochondrial grouping in proximity to the satellite cells and the nucleus of a muscle cell in the MTJ region (Fig. 3H). In the R Group, we discovered the presence of telocytes in proximity to the MTJ interface surrounded by transversal collagen fibrils (Fig. 3C), near the blood capillaries (Fig. 3F), associated with the pericytes. Also, we observed communications between telopodes, cytoplasm of the pericyte and sarcoplasmatic invaginations. Their visible vesicles revealed the paracrine function of telocytes in the MTJ (Fig. 3I).

### Immunofluorescence

**Figure 4:**
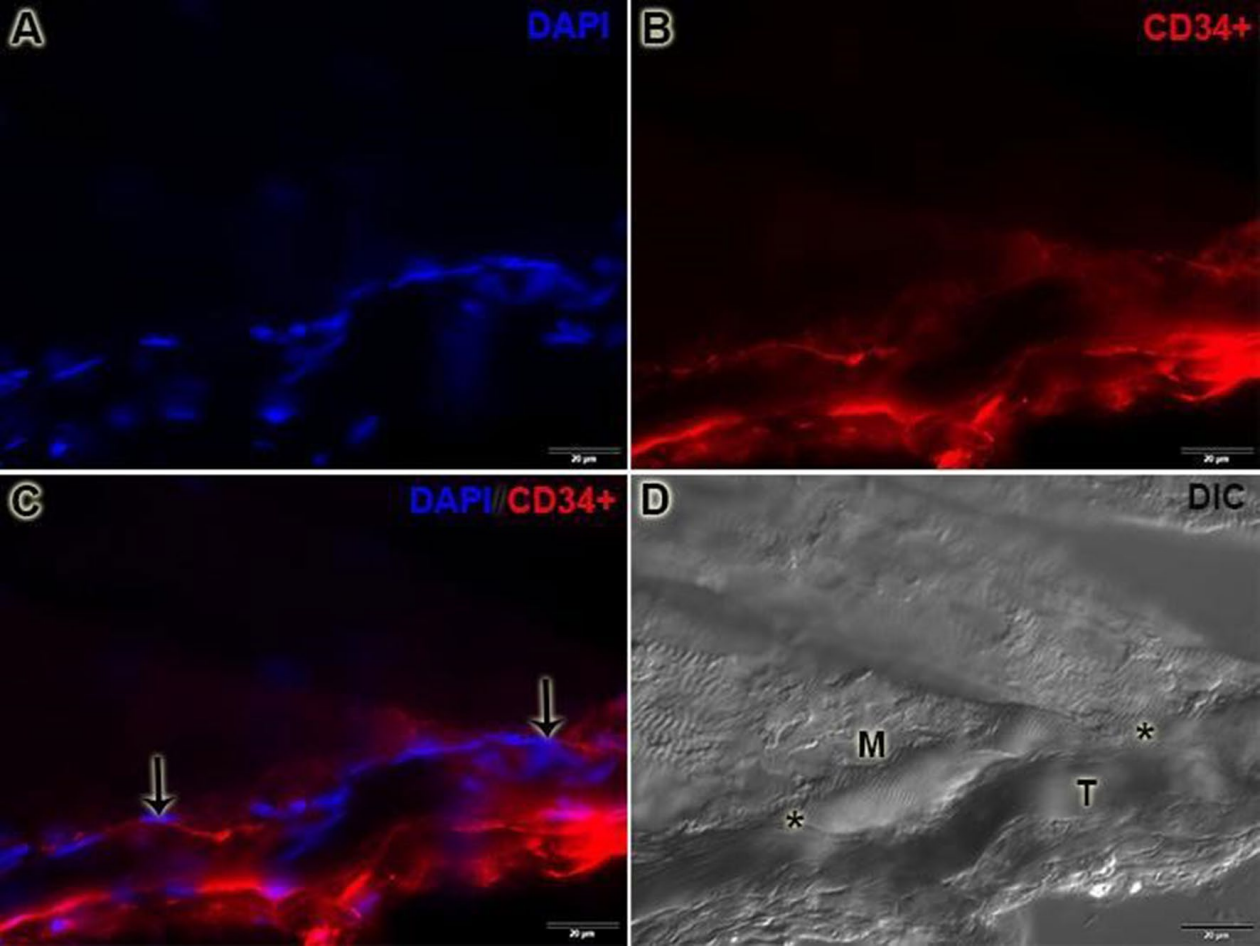
CD34+/Immunostaining is identifying the telocyte niche in the MTJ. A. It's possible to observe the identification of the diversity of nucleus with 4′,6-diamidino-2-phenylindole (DAPI) in this region. B. We identified the telocyte niche in the MTJ and the tendon region with CD34+ immunostaining. C. With the immunostaining, the association is possible to determine the real niche of telocytes in the MTJ D. DIC image to demonstrate the muscle (M) and tendon (T) interaction and the MTJ line (*) between these tissues. Scale Bar: 20 µm. Magnifications: 400× (**A**–**D**).

The CD34+/immunostaining in the MTJ was possible to prove the relationship of telocytes and the MTJ. They are present in the interface of the muscle–tendon region and form a line to the MTJ supporting their structure, corroborating with the results found in the electronic microscopy (Fig. 4).

## Discussion

The different LRT protocols elicited unprecedented results in the structure of MTJ, which showed adaptations in the plantaris and gastrocnemius muscles. Also, we reveal the presence of blood capillaries in the region of the tendon near to the MTJ, provide details on the adaptive responses of the SCL, and observe the first evidence of the existence and micro anatomical location of telocytes between muscle and tendon tissues.

Adaptations found in group C, such as increases in the thickness and length of invaginations and reductions in sarcoplasmatic evaginations, demonstrated that LRT without additional loading might represent an activity that induces MTJ adaptations. On the other side, the more extensive invagination lengths found in group R corroborated the findings using progressive intensity treadmill exercise^24^, indicating a higher adaptive response of muscle tissue in the MTJ, especially for the sarcoplasmatic evaginations.

Resistance exercise promotes morphological adaptations that contribute to alterations of the contact surface of the MTJ, correlating with increases in muscle strength^3,24^. These adaptations corroborate recent studies^25^ that associated such adjustments with the prevention of sports injuries and future traumas resulting from physical inactivity and aging, remodeling their structures in response to different stimuli.

The basal lamina that delimits the ECM in the MTJ exhibited a reduction in thickness in response to the training protocols. In the R group, there was a significant attenuation in thickness when compared to group C. These results differ from those found in studies of treadmill exercise that demonstrated an increase in the thickness of this structure in trained groups^25^. These findings may be associated with metalloproteinases action in this region since there are several levels of laminin and collagen type IV^26^. These elements are responsible for the formation of the basal lamina and are associated with myogenic activity^27,28^.

The observed morphological rearrangement of the basal lamina indicates the activity of the satellite cells that control the homeostatic balance in tissue damage^27^ and promote changes in membrane proteins such as the various types of collagens in this region, which may influence their adaptations, and consequently, the adaptations of adjacent cells^28^.

Satellite cell activity in the MTJ of the trained groups may be associated with specific molecular and cellular adaptations. These results may determine a myoblastic proliferation niche of satellite cells near to the MTJ and the beginning of a post-inflammatory process arising from cell damage^29^, mainly because in this region, the telocytes were also found, reinforcing the niche indication in the MTJ provided.

The adaptation of the supporting collagen layer and the transversely arranged fibrils corroborates the relationship between increased collagen deposition and muscle hypertrophy^30^. Consequently, this fact can promote the remodeling of the ECM and the increase of collagen production in different regions of the MTJ and of the skeletal muscle involved in this type of exercise^31,32^.

The increase in collagen promotes structural contributions and compensation of the ECM with the belly muscle, and based on the contractile transmission force required in this region, it supports a lower risk of injury and increased resistance^33,34^.

The presence of capillaries close to the telocytes is an adjustment that can promote better delivery of nutrients and exchange of gases in this region, allowing paracrine activity between the two structures and better remodeling^12,16,35^. These data indicate one more factor linked to cell damage and, consequently, to muscle regeneration caused by hypertrophic, myogenic, and trophic factors that can act in the MTJ region^34^. The close relationship between telocytes and collagen fibrils was found in MTJ and corroborates with some research in other tissues such as mouse aorta^36^, turtle pancreas^37^, goat rumen smooth muscle layers^12^, and endocardial muscle^38^. Also, such association and communication with the support for these ultrastructures in the interstitial region may be associated with the regeneration and repair of the associated tissue^39,40^.

Also, the identification of a niche of telocytes and their telopodes in MTJ in all groups reveals yet another niche region of these cells and their involvement with other associated cellular structures.

## Conclusion

We concluded that different LRT promoted adaptations in the MTJ as exhibited by the sarcoplasmatic invaginations and evaginations, contributing to an increased contact area in their interface. Still, we identified an SCL adjacent to the MTJ We revealed the first evidence of the existence and location of telocytes in the MTJ inside and adjacent to the SCL, in proximity to capillaries, pericytes, and collagen fibrils. Further studies are needed to investigate these associations and the functions of the telocytes in this central region.

## Materials and methods

### Animals

Thirty *Wistar* rats (90 days) were divided into three groups (n = 10 per group): sedentary (S): not subjected to training protocols; calisthenic (C): subject to ladder-based RT protocol without a load; and resistance-trained (R): subject to ladder-based RT with a progressively increasing weight. The animals were kept in cages at a controlled temperature (23 ± 2 ºC with 12 h light/dark periods) and food and water available ad libitum. All proceedings were approved by the Animal Use Ethics Committee (CEUA)—UNESP (No. 0080) and carried out following the National Council for Animal Experiment Control (CONCEA).

For the RT, a vertical ladder was used (110 cm height, 2 cm between the steps, and 80° inclination). Between climbs, rats remained in a housing chamber (20 cm^3^) at the top of the ladder.

Rats from the C group performed seven unloaded climbs with 1 min intervals between each climb. This protocol was repeated 3×/week for 8 weeks, with a total of 24 training sessions.

For the R group, the rats performed 4 to 9 progressive load climbs equivalent to 50%, 75%, 90%, and 100% of their body mass. From the 5th climb, rats were subjected to 100% load plus 30 g of extra weight by climb until the nine climbs were completed or exhaustion occurred. The protocol was repeated 3×/week for 8 weeks, for a total of 24 training sessions^41^.

### Plantaris muscle mass

The plantaris muscle mass was measured with a semi-analytical balance (Marte Científica AD330) at the end of experimental protocols. After obtaining the data of means ± SD, statistical analysis was performed with Graph Pad Prism 8.0 Software with one-way ANOVA analysis and a Bonferroni post-test (p < 0.05).

### Transmission electron microscopy

Rats (n = 5) were euthanized with an anesthetic overdose (ketamine at 100 mg/kg and xylazine at 5 mg/kg). MTJ samples of the plantaris muscle (3 mm^3^) were immersed in 0.1 M sodium cacodylate solution (pH 7.4) at 4 °C, post-fixed with 1% osmium tetroxide solution^42^, washed with 0.9% saline, and fixed with 0.5% uranyl acetate solution. Then, the samples were dehydrated in a series of alcohols and propylene oxide (2×), followed by a mixture of resin (Spurr) and propylene oxide, and then placement in pure resin for 12 h. The samples were heated at 37 °C in rectangular silicone molds filled with pure resin and kept at 60 °C. Ultrathin (60 nm) sections were collected on 200 mesh copper screens (Sigma-Aldrich, USA), stained with 4% uranyl acetate solution, and after washing, stained with aqueous 4% lead citrate solution 0.4%^43^. The screens were examined with a transmission electron microscope (Philips CM 100, JEOL 1010) at the Institute of Biomedical Sciences of the University of São Paulo, São Paulo-SP, Brazil.

### Immunofluorescence

The MTJ of the gastrocnemius muscle of the experimental groups (n = 5) was dissected and cryofixed in liquid nitrogen − 196 °C. The samples were sectioned longitudinally at 10 µm (Criostaty—HM 505 E, MICROM) and subjected to CD34 immunostaining assays for identifying the telocyte niche in MTJ. After that, the slides were washed in phosphate-buffered saline (PBS) for 5 min and incubated overnight at 4 °C with primary antibody CD34 (1:1,000, IgG polyclonal, Invitrogen, PA5-85917) diluted in PBS with 1% bovine serum albumin (BSA). After two washes in PBS, the slides were incubated with goat anti-rabbit secondary antibody Alexa Fluor 594 (1:1,000, IgG, Invitrogen, A-11012) diluted in PBS with 1% BSA for 1 h at room temperature. Nuclei were stained with 4′,6-diamidino-2-phenylindole (DAPI) (Molecular Probes, Eugene, P36935). Histological sections were analyzed with a fluorescence microscope (Olympus X61). Differential interference contrast image was captured with a magnification of 200× to visualize the muscle fiber disposition and interface muscle–tendon.

### Morphometric analysis

We used the plantaris muscle to made all the measurements.

The evagination and invagination lengths measured at the basement of the MTJ to the apical point of ultrastructures (number of ultrastructures measured per group = 70). The thicknesses made from the MTJ ultrastructures (number of ultrastructures measured per group = 70). The basal lamina thickness measurement was at the MTJ interface at the final line of the basal lamina (number of ultrastructures measured per group = 50). The SCL thickness measurement was at the MTJ interface at the last line formed by multiples transverse collagen fibrils adjacent to the MTJ (number of ultrastructures measured per group = 50) represented in Fig. 2. The diameter of the collagen fibrils made in the fibrils that form the SCL adjacent to the MTJ (number of ultrastructures measured per group = 30) represented in Fig. 2. ImageJ software used to measure all the data. After obtaining the data of means ± SD, statistical analysis performed with Graph Pad Prism 8.0 Software with one-way ANOVA analysis and a Bonferroni post-test (p < 0.05).

## Data Availability

All relevant data are within the paper.
